# The surface phase diagram of Fe_3_O_4_(001) revisited†

**DOI:** 10.1039/d5lf00022j

**Published:** 2025-03-14

**Authors:** Panukorn Sombut, Matthias Meier, Moritz Eder, Thomas Angerler, Oscar Gamba, Michael Schmid, Ulrike Diebold, Cesare Franchini, Gareth S. Parkinson

**Affiliations:** a Institute of Applied Physics, TU Wien Vienna Austria parkinson@iap.tuwien.ac.at; b Faculty of Physics, Center for Computational Materials Science, University of Vienna Vienna Austria; c GeoRessources, Université de Lorraine, CNRS 54000 Nancy France; d Dipartimento di Fisica e Astronomia, Università di Bologna Bologna Italy

## Abstract

Understanding how the physical and electronic structures of metal-oxide surfaces evolve under varying conditions is crucial for optimizing their performance in applications such as catalysis. In this study, we compute the surface phase diagram of the Fe_3_O_4_(001) facet using density functional theory (DFT)-based calculations, with an emphasis on understanding the terminations observed in surface science experiments. Our results reveal two stable terminations in addition to the subsurface cation vacancy (SCV) structure, which dominates under oxidizing conditions. The commonly reported octahedral Fe pair, also known as the Fe-dimer termination, is stable within an oxygen chemical potential range of −3.1 eV < *μ*_O_ < −2.3 eV. We identify the lowest-energy structure of this surface as the one proposed by J. R. Rustad, E. Wasserman and A. R. Felmy, A Molecular Dynamics Investigation of Surface Reconstruction on Magnetite (001), *Surf. Sci.*, 1999, **432**, 1–2, where a tetrahedrally coordinated Fe_A_ atom is replaced by two octahedrally coordinated Fe_B_ atoms in the surface layer. This transformation serves as a precursor to the emergence of an FeO-like termination under highly reducing conditions. A key insight from our study is the importance of thoroughly sampling different charge-order configurations to identify the global minima across varying stoichiometries.

## Abstract 2

Understanding how the physical and electronic structures of metal-oxide surfaces evolve under varying conditions is crucial for optimizing their performance in applications such as catalysis.

## Graphical abstract

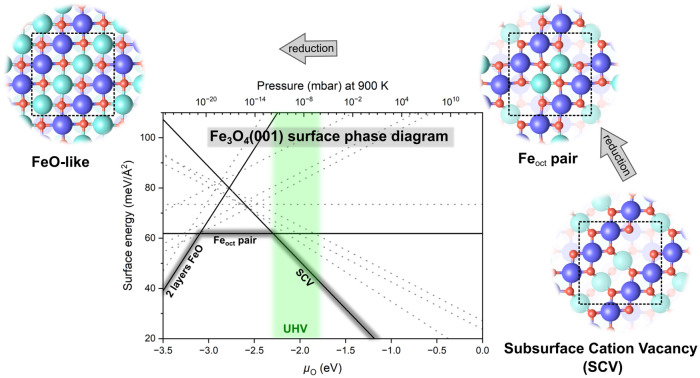

## Introduction

**Fig. 1:**
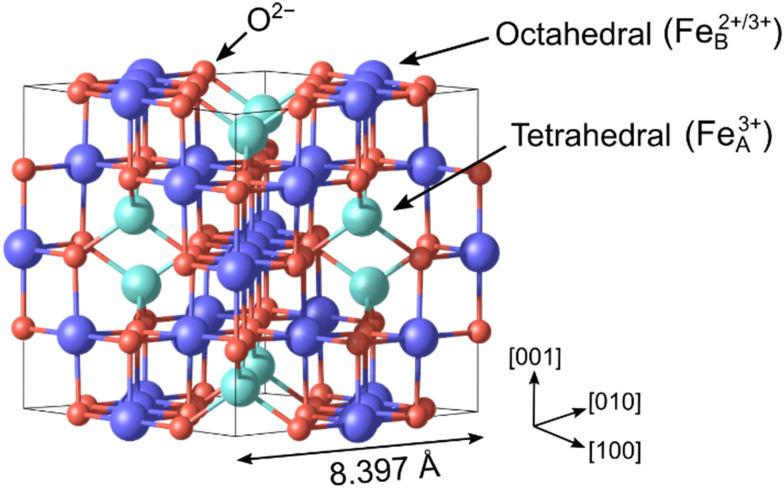
The bulk structure of Fe_3_O_4_ at low temperature is based on an fcc arrangement of O^2−^ cations (red), where Fe^3+^ cations occupy tetrahedral sites (Fe_A_, cyan), and a 1 : 1 mixture of Fe^2+^ and Fe^3+^ cations resides in octahedral sites (Fe_B_, dark blue). The experimental lattice parameter at room temperature is 8.397 Å.

Iron, one of the most abundant elements in the Earth's crust, undergoes oxidation in the ambient atmosphere to form various oxides and hydroxides.^1^ Hematite (α-Fe_2_O_3_), the most stable oxide phase under oxidizing conditions, has a corundum structure with Fe^3+^ cations located in octahedral interstitial sites of a hexagonal close-packed (hcp) O^2−^ lattice. Wüstite (Fe_1−*x*_O) forms under reducing conditions; this material takes the rocksalt structure with Fe^2+^ cations occupying octahedrally coordinated sites in the face-centered cubic (fcc) O sublattice. Magnetite (Fe_3_O_4_) crystallizes in an inverse spinel structure (AB_2_O_4_), where Fe^3+^ cations occupy tetrahedral sites (A sites) and a 1 : 1 mixture of Fe^2+^ and Fe^3+^ cations reside in octahedral sites (B sites),^2^ as seen in Fig. 1. The Fe_A_ and Fe_B_ cations have antiparallel spin orientations, making Fe_3_O_4_ a ferrimagnet. Above the Verwey transition temperature of 125 K, the Fe_B_ atoms become equivalent and have a nominal Fe^2.5+^ charge state.^3^ This results in room-temperature half-metallicity,^4^ which is potentially useful for spintronics applications.^5,6^ The redox properties of iron oxides^7^ make their surfaces important in geochemistry^1^ and there is a long-standing interest in understanding how the transformation between phases begins at the surface and propagates through the material. Finally, iron oxides are important in catalysis,^1,8–11^ both as a reducible support for precious metals and as the active phase for reactions such as the high-temperature water-gas shift reaction.^12,13^

Over recent years, the Fe_3_O_4_(001) surface has emerged as an ideal model system to study fundamental processes occurring at Fe oxide surfaces. In contrast to other much-studied facets such as Fe_3_O_4_(111) and α-Fe_2_O_3_(0001),^14–16^ a monophase termination is straightforward to prepare on Fe_3_O_4_(001) under ultrahigh vacuum conditions (UHV). The most commonly observed surface has a 
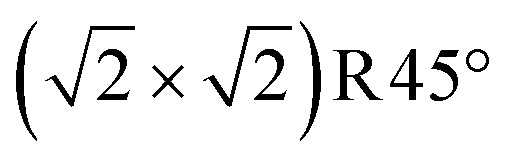
 periodicity based on an array of subsurface cation vacancies and interstitials. This “subsurface cation vacancy” (SCV) structure was determined using a combination of scanning tunneling microscopy (STM),^17^ quantitative low-energy electron diffraction (LEED),^17^ surface X-ray diffraction (SXRD)^18^ and density functional theory (DFT).^17^ Angle-resolved XPS measurements confirm it to be oxidized with respect to the bulk spinel structure.^17^ When Fe_3_O_4_(001) samples are reduced, STM images reveal an alternative termination featuring pairs of protrusions with a 
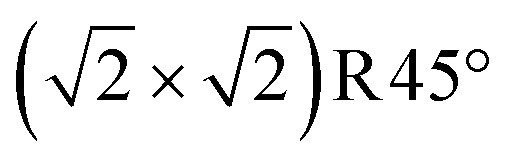
 periodicity. This is known as the octahedral Fe pair termination (hereafter Fe_oct_ pair termination), or the Fe-dimer termination.^19–25^ The Fe_oct_ pair termination was experimentally observed by many groups, on single crystals subjected to many sputter/anneal cycles, after Fe deposition on Fe_3_O_4_(001) bulk crystal,^25,26^ or on epitaxial thin films containing an excess of Fe.^20,27^ Several models have been proposed,^20,26,28^ which differ in stoichiometry and how the Fe atoms are positioned with respect to the subsurface.

In this study, we use first-principles calculations based on DFT to revisit the surface phase diagram of Fe_3_O_4_(001). We demonstrate that the model originally proposed by Rustad *et al.*^28^ represents the most stable variant of the Fe-octahedral (Fe_oct_) pair termination. Compared to the B-terminated truncated bulk, this structure replaces one Fe_A_ atom with two octahedrally coordinated Fe atoms in each reconstructed 
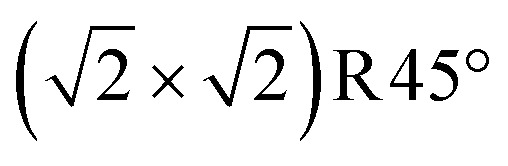
 unit cell. It is stoichiometric, charge-neutral, and satisfies polarity compensation requirements within a purely ionic framework. Under extremely reducing conditions, an FeO-like termination emerges, effectively propagating Rustad's mechanism across the surface. Furthermore, our calculations reveal that the spin orientation and the charge ordering between Fe^2+^-like and Fe^3+^-like ions, particularly in the topmost surface layer, play a crucial role in determining surface stability.

## Computational details

The Vienna *ab initio* simulation package (VASP)^29^ was used for all calculations, with the near-core regions described by the projector-augmented-wave method.^30,31^*Γ*-Centered *k*-meshes of 5 × 5 × 5 and 3 × 3 × 1 were used for the bulk optimization (*Fd*3̄*m*) and all surface slabs, respectively, and the plane-wave basis set cutoff was set to 550 eV. Calculations were performed using the regularized-restored strongly constrained and appropriately normed meta-generalized gradient approximation (r^2^SCAN)^32^ with an on-site Coulomb repulsion term^33^*U*_eff_ = 3.10 eV for the 3d electrons of the Fe atoms,^34^ where the magnitude of *U* is determined from experimental oxidation energies. In accordance with the experimental conditions, the bulk lattice constant was optimized for the room-temperature phase (Fe^2.5+^) by enforcing *Fd*3̄*m* symmetry of the charge density. All other calculations were conducted using the electronic ordering of the low-temperature phase, as there was no straightforward method to stabilize the room-temperature phase and prevent charge disproportionation into Fe^2+^ and Fe^3+^ on an arbitrary surface with broken symmetry. In addition to the r^2^SCAN calculations, we also adopted the generalized gradient approximation method with the Perdew–Burke–Ernzerhof (PBE) functional,^35^ with an effective on-site Coulomb repulsion term *U*_eff_ = 3.61 eV.^36,37^ The PBE-optimized bulk overestimates the lattice constant by ≈1%. The r^2^SCAN performs better by only overestimating it by ∼0.2%. The calculations were also performed with the experimental magnetite lattice parameter (*a* = 8.397 Å),^38^ which may provide increased accuracy with PBE+U in some cases.^39^ The relative surface energies exhibited minor changes, but these did not significantly alter the conclusions regarding the surface phase diagram of Fe_3_O_4_(001). A symmetric slab was built with 17 planes (9 planes with octahedral Fe and 8 with tetrahedral Fe; only one Fe atom in the middle layer is fixed, and the rest relaxed) and 14 Å vacuum. The convergence criterion was an electronic energy step of 10^−6^ eV and forces acting on ions smaller than 0.02 eV Å^−1^. Simulated scanning tunneling microscopy (STM) images were generated using the Tersoff–Hamann approximation in constant-height mode, allowing visualization of local electronic density at specific energy levels for a sample bias voltage of +2.5 V.^40^ Surface phase diagrams were derived following the approach described by Reuter and Scheffler.^41^ We note that the symmetry of the surface slab is broken; therefore the bulk energy reference of Fe_3_O_4_ was calculated without enforcing cubic symmetry (resulting in the *P*/2*c*-like low-temperature phase).^42^
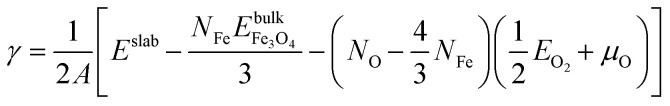
Here, *γ* is the surface energy, and 2*A* is the surface area of the slab (two sides). *E*^slab^ is the total energy of the surface structure obtained from DFT calculations. 
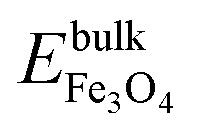
 is the energy for one formula unit of the bulk (*P*/2*c*), and 
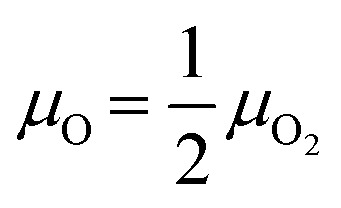
, where *μ*_O_2__ is the chemical potential of oxygen in the gas phase. *E*_O_2__ is the total energy of an isolated O_2_ molecule in a spin-polarized calculation at *T* = 0 K. *N*_Fe_ and *N*_O_ are the numbers of Fe and O atoms in the surface slab, respectively. The chemical potential of oxygen in the gas phase provides the temperature and pressure dependence in the phase diagram and can be calculated as
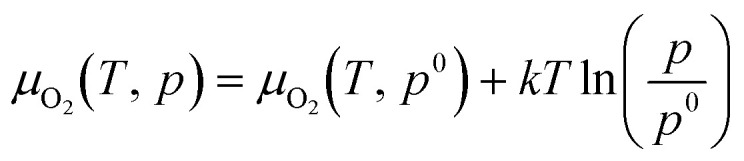
where *p*^0^ is a reference pressure and *μ*_O_2__(*T*, *p*^0^) = *H*(*T*, *p*^0^) − *TS*(*T*, *p*^0^) can be calculated from tabulated data.^43^

To check for possible errors in computing the energy of O_2_ by DFT, we have calculated the formation energy of bulk Fe_3_O_4_ using the r^2^SCAN functional, using O_2_ and metallic Fe bulk (BCC) as a reference. Since metallic Fe cannot be correctly described by DFT+U, this was done with *U* = 0. Our calculated formation energy is −11.70 eV, compared to an experimental value of −11.55 eV at 0 K.^43^ This results in an estimated error of −0.04 eV per O atom. Previous studies have reported somewhat larger discrepancies. Almeida *et al.* found an error of −0.23 eV per O atom for the SCAN functional,^44^ while Hütner *et al.* estimated a comparable error of −0.20 eV per O atom for r^2^SCAN.^45^ In any case, it is important to note that the O_2_ binding energy error causes a minor shift in the *μ*_O_ axis of the phase diagrams, but it does not alter the relative ordering of the compared phases.

To selectively control the charge ordering of Fe^2+^ and Fe^3+^ ions on the surface layer, we used the occupation matrix control tool,^46^ which consists of an initial constrained calculation (with an input occupation matrix kept fixed during the calculation) followed by an unconstrained calculation. In what follows, Fe^2+^-like and Fe^3+^-like cations are identified by the calculated Bader charges^47^ (Tables S1–S7†) of 1.30–1.45 *e* and 1.70–1.80 *e*, respectively, and the local magnetic moments. Magnetic orientation also plays a crucial role in surface stability. Therefore, we also explore the magnetic ordering and the charge ordering of various Fe_3_O_4_(001) terminations in reducing conditions.

## Results

### I. Surface phase diagram

**Fig. 2:**
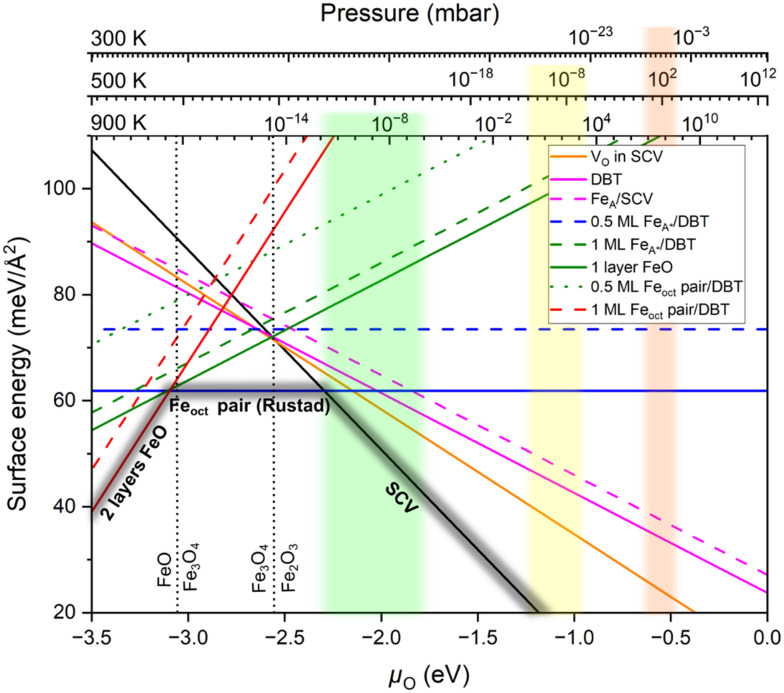
Surface phase diagram of Fe_3_O_4_(001) comparing the surface energies of various terminations as a function of oxygen chemical potential (*μ*_O_); derived from r^2^SCAN+U calculations. The top axes indicate the corresponding oxygen partial pressures at 300 K, 500 K, and 900 K.^43^ The light orange, light yellow, and light green regions indicate the range of UHV experiments (10^−12^ mbar < *p*_O_ < 10^−6^ mbar) corresponding to each temperature: 300 K, 500 K, and 900 K respectively. Dashed vertical lines show the calculated Fe_2_O_3_(hematite)–Fe_3_O_4_(magnetite) and Fe_3_O_4_(magnetite)–FeO(wüstite) phase equilibrium. The convex hull formed by the three stable terminations is highlighted in dark gray.

Fig. 2 presents our surface energy diagram of Fe_3_O_4_(001). To the best of our knowledge, this is the most comprehensive diagram of this type for Fe_3_O_4_(001) published since the discovery of the SCV reconstruction.^2,17^ In particular, we focus on understanding the structures observed experimentally in surface science investigations under reducing conditions. We note that Fe_3_O_4_(100), Fe_3_O_4_(010) and Fe_3_O_4_(001) are equivalent above the Verwey transition. However, the DFT calculations were performed at 0 K, which is below the Verwey transition, where the phase becomes monoclinic due to the charge ordering between Fe^2+^ and Fe^3+^ ions. As a result, the (110), (010), and (001) faces are no longer equivalent. However, this difference is minimal, and we therefore expect similar results for the Fe_3_O_4_(100) and Fe_3_O_4_(010). This diagram was calculated at the r^2^SCAN+U level (Fig. S8† shows an alternative diagram based on PBE+U calculations) and derived from the framework of *ab initio* atomistic thermodynamics.^41^ The upper *x*-axes show the corresponding oxygen partial pressures at three different temperatures: 300 K (room temperature), 500 K (the minimum temperature at which the Fe diffusion occurs^48^), and 900 K (the typical annealing temperature in UHV experiments). Three surface structures were identified as stable across the considered oxygen chemical potential range: (1) the SCV termination,^17^ (2) the Fe-octahedral (Fe_oct_) pair termination, and (3) a reduced FeO-like termination. In what follows, we discuss the various structures, including those that do not appear in the convex hull, starting with the SCV termination. This choice is partly motivated by experiments where Fe was systematically deposited on the SCV termination and the resulting structures imaged with STM.^19,26^

### II. Oxidized surfaces

**Fig. 3:**
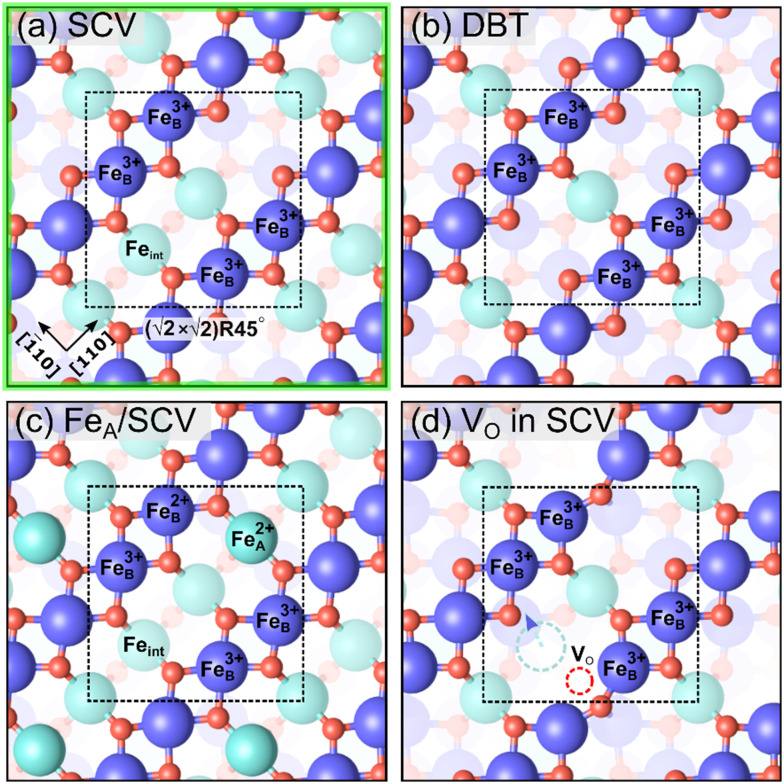
Non-stoichiometric (oxidized) terminations of the Fe_3_O_4_(001) surface (top view). Iron is large and blue or cyan, and oxygen is small and red. Dark blue (spin up) and cyan (spin down) indicate the spin orientation in Fe atoms, which coincide with the Fe_B_ and Fe_A_ sublattices, respectively. The 
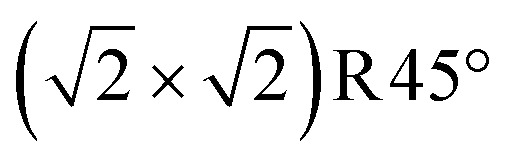
 cell is indicated with a black dashed square. (a) SCV termination, (b) distorted B termination (DBT), (c) Fe_A_ adatom on SCV, and (d) V_O_ in SCV, where the oxygen vacancy is shown as a red dashed circle. With respect to a stoichiometric termination, SCV, DBT, Fe_A_ on SCV, and V_O_ in SCV terminations have a deficiency of 2, 1, 1, and 1.25 Fe atoms per 
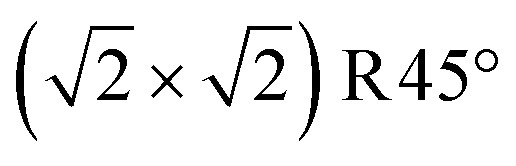
 cell, respectively. The highlighted green square indicates that (a) is one of the three stable terminations in the surface phase diagram of the Fe_3_O_4_(001).

We begin with the non-stoichiometric subsurface cation vacancy (SCV) termination (Fig. 3a), which has been described extensively in the past.^17,18^ Compared to a bulk truncation at the Fe_B_–O plane (Fig. 3b), two Fe_B_ cations from the third layer are removed and one Fe_A_ cation is added in the second layer. The Fe interstitial (labelled Fe_int_ in Fig. 3a) has a Bader charge of 1.75 *e*, and is thus Fe^3+^-like, like all the other tetrahedrally coordinated Fe atoms. The Bader charges indicate that all Fe atoms in the outermost four layers are Fe^3+^ (Table S1†), which is explicable since the surface is oxidized with respect to bulk Fe_3_O_4_ (2 Fe atoms missing per 
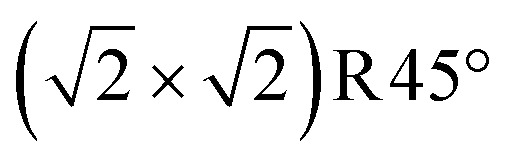
 unit cell, compared with a stoichiometric termination). We compute the SCV termination to be the most favorable surface for O_2_ chemical potentials greater than −2.30 eV (black line in Fig. 2).

Introducing one additional Fe atom to the SCV structure leads to two possible surface terminations: the distorted bulk truncation (DBT) (alternatively named the distorted Fe_B_–O termination) originally proposed by Pentcheva and coworkers^49^ (Fig. 3b), and a surface with an Fe adatom on the SCV-reconstructed surface (Fe_A_ on SCV) (Fig. 3c). The DBT exhibits a 
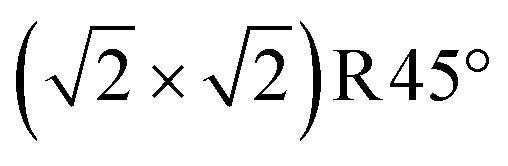
 periodicity due to a Jahn–Teller effect, in which a lattice distortion is coupled to subsurface charge order.^49–51^ This structure was further discussed in terms of a surface Verwey transition,^52^ and proposed to explain the undulation of the surface Fe_B_ rows observed in STM images.^53,54^ However, this model ultimately failed to explain some properties of the experimentally observed 
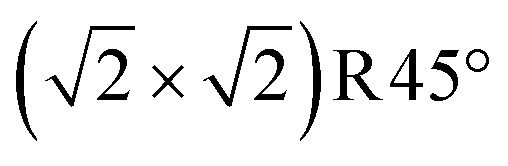
 surface, most notably the preference for metal adatoms to bind at one particular site in the 
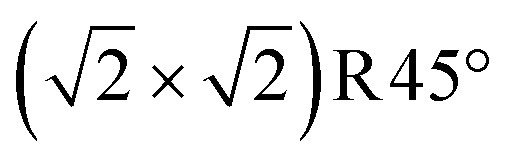
 unit cell.^55–57^ In the DBT model, when ignoring the small distortions, there are two equivalent adsorption sites per 
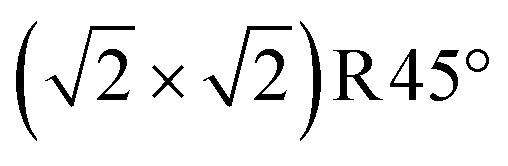
 unit cell; these sites correspond to the tetrahedral positions of the next layer when continuing the bulk lattice. In the SCV model, one of these sites is blocked by the Fe_int_, thus there is only one site for metal adatoms without a tetrahedral Fe_A_ below.

When an Fe adatom is placed on the SCV termination (Fig. 3c), it binds to the two oxygen atoms that do not have a subsurface Fe_A_ neighbor. The optimized structure obtained by r^2^SCAN+U reveals the surface Fe adatom relaxes downward from the tetrahedral bulk-continuation Fe_A_ site due to its low (twofold) coordination with oxygen. Whereas a bulk-like Fe_A_ atom is Fe^3+^, the surface Fe_A_ adatom has a Bader charge of 1.34 *e* and is thus Fe^2+^. Previous STM studies have identified Fe_A_ adatoms as a defect on the as-prepared SCV surface,^48^ but a complete monophase 0.5 ML Fe_A_ termination was never observed. These experimental results are in line with our calculations, which determine that neither DBT nor Fe_A_ on SCV is favored in the surface phase diagram at any O chemical potential.

Since reduction can also occur through an oxygen vacancy (V_O_), we also computed the presence of oxygen vacancies in the surface layer (Fig. 3d). The oxygen atoms that can be removed at the lowest cost are those binding to the Fe_int_ in the SCV termination. In the optimized structure, the Fe_int_ from the SCV reconstructed surface moves downward to occupy one of the third-layer Fe_B_ vacancy sites (cyan dashed arrow and cycle in Fig. 3d). This Fe atom prefers the site opposite to the oxygen vacancy rather than directly beneath it. The oxygen vacancy in SCV (V_O_ in SCV) structure is more stable than the DBT and Fe_A_ on SCV terminations for oxygen chemical potentials greater than −2.66 eV (Fig. 2). Nevertheless, this structure is not on the convex hull and, thus, not present at the surface under equilibrium conditions.

### III. Stoichiometric surfaces

**Fig. 4:**
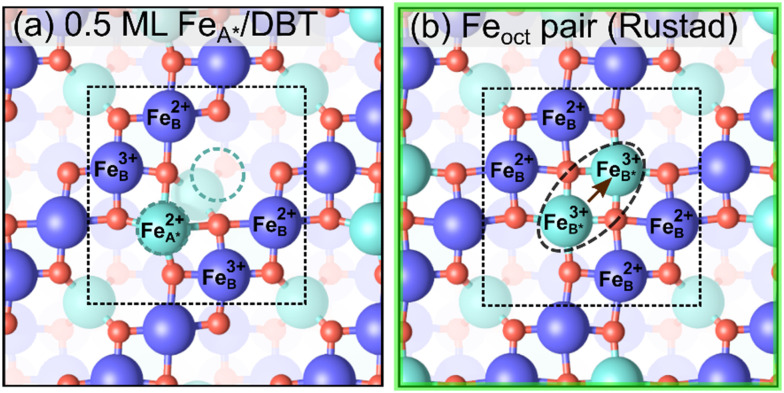
Stoichiometric terminations of the Fe_3_O_4_(001) surface (top view). These structures contain 2 additional Fe atoms per unit cell with respect to the SCV surface. Iron is large and dark blue (spin up) or cyan (spin down), and oxygen is small and red. Fe atoms are labelled with their charge state inferred from the Bader charges (≈1.77 *e* and 1.40 *e* for 3+ and 2+, respectively). The 
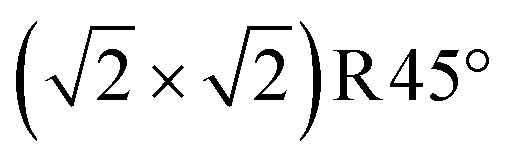
 cell is indicated with a black dashed square. (a) 0.5 ML of Fe_A*_ on DBT, and (b) Fe_oct_ pair termination originally proposed by Rustad.^28^ The black dashed oval indicates the octahedral pair. The brown arrows and dotted cyan circles show that 0.5 ML Fe_A*_ surface can be converted into the Fe_oct_ pair by moving two tetrahedral Fe atoms. The green square indicates that the structure in panel (b) is one of the three stable terminations in the surface phase diagram.

Next, we explore the stoichiometric surface terminations of Fe_3_O_4_(001), featuring two additional Fe atoms with respect to the SCV structure in a 
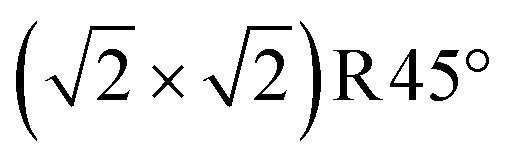
 periodicity. One possible structure model with this composition involves an Fe adatom on a surface truncated at the Fe_B_–O plane (Fig. 4a). Previous authors have referred to this as the 0.5 ML Fe_A_ termination,^23,25,58^ because one of the two equivalent Fe_A_ bulk continuation sites is occupied. In contrast to Fe_A_ sites of the bulk, this Fe_A_ atom does not have tetrahedral but only twofold coordination. Upon relaxation, our DFT calculations reveal that the Fe_A_ does not remain centered at the 2-fold coordinated site, but increases its oxygen coordination by a horizontal shift, which puts it close to an octahedral site. Since it resides rather far (1.11 Å) from the tetrahedral site, we name it Fe_A*_. The relaxation is limited by repulsion between the Fe_A*_ and one of the Fe_A_ atoms in the first subsurface layer (at a distance of only 2.53 Å). The energy gain of this relaxation is 4.62 meV Å^−2^ (Fig. S1†). Therefore, we call this the “0.5 ML Fe_A*_” termination. Pentcheva and coworkers observed similar behavior of 0.5 ML and 1 ML of Co adatoms on the Co_3_O_4_(001) surface.^59^ We consider this structure an initial state, transitioning to the more favorable the Fe_oct_ pair termination proposed by Rustad *et al.*^28^

Rustad's Fe_oct_ pair termination^28^ (Fig. 4b) is not only energetically superior to the 0.5 ML Fe_A*_ on DBT by 11.62 meV Å^−2^ (equivalent to 0.82 eV per 
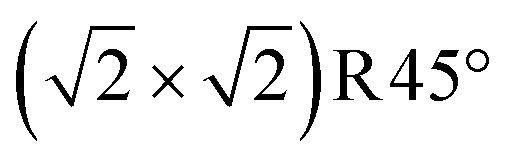
 surface cell), but it is also the most preferred structure for a stoichiometric surface. Furthermore, the termination also lies on the convex hull in the phase diagram (Fig. 2). The stability range of this structure is −3.10 eV < *μ*_O_ < −2.30 eV. We have also tried an additional stoichiometric termination, an Fe_oct_ pair on the SCV termination; this is substantially worse in energy. Rustad's Fe_oct_ pair structure was proposed based on molecular dynamics simulations, which showed that the surface Fe_A_ adatom moved laterally and downward to occupy an octahedrally coordinated site (Fe_B*_) in the surface Fe_B_–O plane. Fe–Fe repulsion then caused a subsurface Fe_A_ atom to move laterally away and upwards (brown arrow in Fig. 4b), and this atom ultimately occupied a similar site with five-fold coordination to oxygen. We will argue here that this is the Fe_oct_ pair termination (or Fe dimer), frequently observed in STM investigations.^19–25^ Moreover, we analyse the magnetic orientation and charge order of the Fe_oct_ pair termination. Our r^2^SCAN+U calculations reveal that the spins of the Fe_oct_ pair couple antiparallel to the Fe_B_ atoms in the surface layer. In this sense, it constitutes a first step towards the wüstite structure of FeO, in which the (001) planes consist of rows with antiparallel spin. In the Fe_oct_ pair termination, two of the oxygen atoms in the surface layer have no Fe neighbor in the layer below, resulting in a 4-fold planar coordination and causing the surface to buckle slightly. In the optimal configuration (Fig. 4b), the Fe_oct_ pair has a Bader charge of 1.77 *e*, which corresponds to Fe^3+^. However, four Fe_B_ atoms in the surface layer are reduced from Fe^3+^ to Fe^2+^ (Bader charges around 1.40 *e*). All four Fe_B_ atoms in the first subsurface layer are Fe^3+^, based on Bader charge analysis and the local magnetic moment of Fe atoms. We note that the r^2^SCAN+U and PBE+U calculations can get stuck in local minima (Fig. S3c†) where the Fe_oct_ pair is Fe^2+^ and four Fe_B_ atoms in the surface layer are mixed between Fe^2+^ and Fe^3+^ ions. This is 7.23 meV Å^−2^ (0.51 eV per 
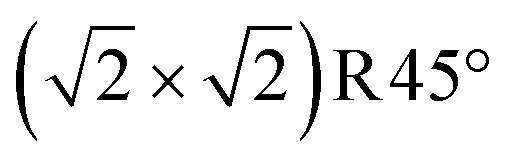
 surface cell) less stable than the electronic ground state (Fig. 4b) at the r^2^SCAN+U level. Different possibilities for the charge order in the surface layer are shown in Fig. S3,† together with their relative energy differences with respect to the optimal electronic configuration. We also tested the hybrid functional HSE06; also with this functional the resulting configurations remained trapped in local minima. To counteract this, we initiated the different initial charge configurations using the occupation matrix tool, which controls the charge order of the Fe atoms in the surface layer. Rustad and co-workers treated the Fe ions as a nominal charge state of +2.5 in their molecular dynamics simulations;^28^ thus, these calculations were not affected by the problem of the charge order.

### IV. Reduced surfaces

**Fig. 5:**
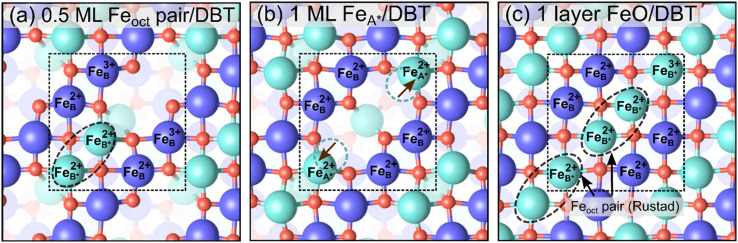
Non-stoichiometric (reduced) terminations of the Fe_3_O_4_(001) surface, which contain 3 additional Fe atoms per 
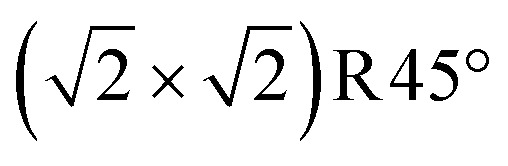
 cell with respect to the SCV surface, one more than the stoichiometric termination (top view). Iron is large and dark blue (spin up) or cyan (spin down), and oxygen is small and red. Black dashed ovals indicate the positions of Fe_oct_ pairs. A 
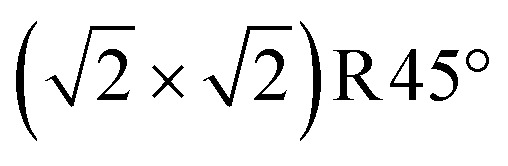
 cell is indicated with a black dashed square. (a) 0.5 ML Fe_oct_ pair on DBT proposed by Novotny *et al.*^26^ (b) 1 ML of Fe_A*_ on DBT, and (c) 1 layer FeO-like on DBT. The brown arrows and dotted cyan circles indicate the Fe_A_ atoms that have relaxed outward from a 2-fold coordination with the oxygen atoms.

Now switching to Fe-rich, non-stoichiometric terminations, Fig. 5a shows the Fe_oct_ pair termination proposed by Novotny *et al.*,^26^ which requires the addition of three Fe atoms per unit cell to the SCV termination (one to lift the SCV reconstruction and restore a bulk truncated surface, and the other two for the additional Fe_oct_ pair itself). This corresponds to one Fe atom per 
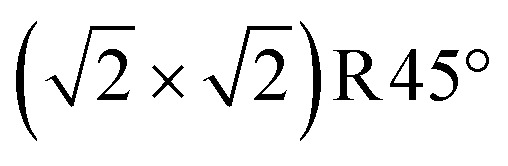
 cell more than the stoichiometric terminations of Fig. 4. The surface energy diagram indicates that this termination (dotted green line in Fig. 2) is energetically less favorable compared to the other terminations proposed here across the entire range of oxygen chemical potential. This is because the added Fe_B*_ atoms and Fe_A_ atoms below are in close proximity, with Fe–Fe distances of only 2.5 Å. The other two terminations with the same, reduced stoichiometry are 1 ML Fe_A*_ (as previously reported by many groups,^26,49,52,60^Fig. 5b) and Fe_oct_-only (Fig. 5c).

When adding two Fe_A_ adatoms to the DBT structure, Fig. 5b shows that these two adatoms again relax from their twofold high-symmetry positions, increasing their oxygen coordination but putting them close to the Fe_A_ atoms in the subsurface (≈2.5 Å). Since the adatoms leave the tetrahedral site, we name them Fe_A*_. In principle, there are two possibilities for such a relaxation, the two Fe_A*_ adatoms of the 
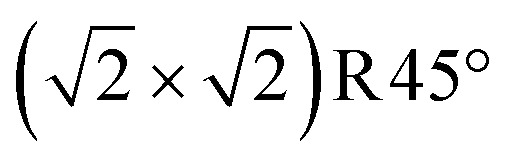
 cell can relax in either the same direction or opposite directions (Fig. S2†); the latter is more favorable. This observation is in line with previous work from Spiridis and coworkers, which suggested that the shifting of Fe_A_ adatoms towards each other might be responsible for the Fe “dimer” surface observed in STM experiments.^20,61^

In Fig. 5c, the two Fe_A_ atoms in the immediate subsurface layer are removed and, together with the Fe_A*_ atoms, from two surface Fe_oct_ pairs. This termination utilizes the mechanism proposed by Rustad^28^ twice in the 
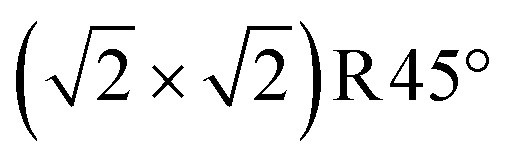
 unit cell. This structure can alternatively be seen as one layer of FeO(001) supported on Fe_3_O_4_(001) terminated at the Fe_B_–O plane (apart from the slight distortions, the DBT surface of Fig. 3b). In the relaxed structure, the four oxygen atoms in the 
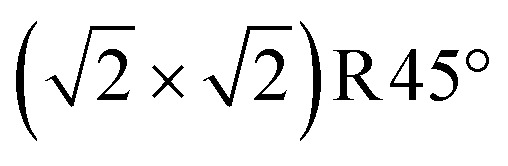
 unit cell that have no Fe neighbor in the subsurface layer relax upward from the surface by around 0.4 Å above the Fe_B_–O surface plane (perspective view in Fig. S5i†). Apart from the vertical buckling, the rows of O atoms also exhibit slight in-plane displacements, which can be also seen in Fig. S5i.† We note that the in-plane displacements of O atoms are due to the orbital interaction between the p-orbitals of the O atoms and the t_2g_ orbitals of the Fe^2+^ ions (see Fig. S7d†).

In the phase diagram (Fig. 2), neither of these reduced terminations is on the convex hull. Concerning the relative stability, r^2^SCAN+U predicts the FeO monolayer (two Fe_oct_ pairs) in Fig. 5C to be lower in energy by 3.31 meV Å^−2^, whereas the 1 ML Fe_A*_ on DBT of Fig. 5b is preferred in a PBE+U calculation. Tests with different charges for the Fe atoms show that this difference between the r^2^SCAN+U and PBE+U is not due to a local energy minimum but indeed caused by the functional. In this respect, it is noteworthy that r^2^SCAN+U predicts a larger energy advantage for the Fe_oct_ pair than PBE+U also for the stoichiometric termination. In any case, charge order plays a significant role in the surface stability of the 1 layer FeO-like termination (Fig. S4†). The surface becomes increasingly stable as the number of Fe^2+^ ions in the surface increases (which increases the number of Fe^3+^ in the first subsurface octahedral layer; this layer converts to fully Fe^3+^ at the favorable charge order of Fig. 5c). Our calculations at the r^2^SCAN+U level reveal that the maximum number of Fe^2+^ ions in the surface layer is that of Fig. 5c, seven Fe_B_ atoms within the 
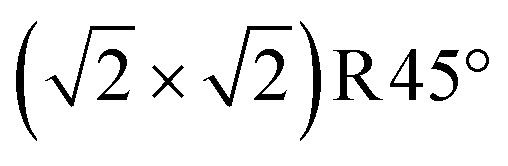
 unit cell.

**Fig. 6:**
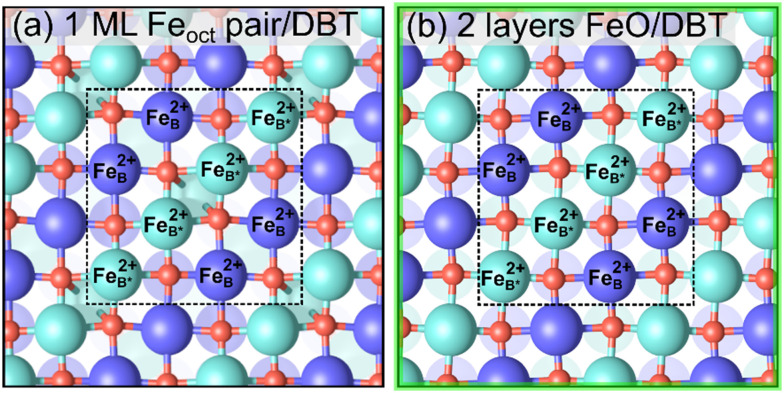
Non-stoichiometric (reduced) terminations of the Fe_3_O_4_(001) surface, which contain 5 additional Fe atoms per 
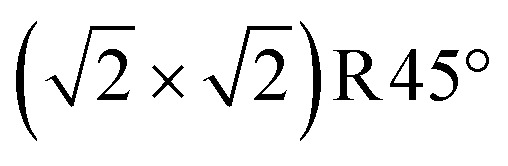
 cell with respect to the SCV surface, three more than the stoichiometric surface (top view). Iron is large and dark blue (spin up) or cyan (spin down), and oxygen is small and red. The 
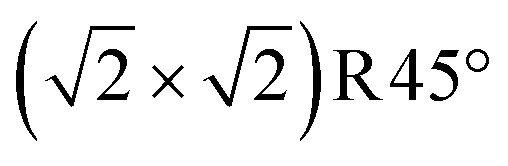
 cell is indicated with a black dashed square. In both structures, all surface iron atoms are Fe^2+^ according to the Bader charges (between 1.34–1.36 *e*). (a) 1 ML Fe_oct_ pair on DBT, and (b) 2 layers FeO-like on DBT. With respect to a stoichiometric termination, these two terminations each contain three additional Fe atoms per 
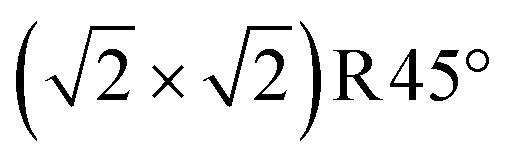
 cell. The highlighted green square indicates that this is one of the three stable terminations in the surface phase diagram of the Fe_3_O_4_(001).

Finally, we examine the surface terminations resulting from the deposition of five additional Fe atoms onto the SCV termination. Previous experiments indicate that an FeO(001)-like surface layer is formed in extremely reducing conditions.^26,62^ In Fig. 6, we propose two possible FeO-like surface terminations: (a) adding 4 Fe_oct_ atoms to the DBT surface without removal of the tetrahedral atoms, and (b) 2 layers FeO-like on the DBT surface with the tetrahedral Fe removed. The latter structure is equivalent to adding one layer of FeO on the all-octahedral surface of Fig. 5c. This can also be viewed as the reduction mechanism proposed by Rustad^28^ occurring four times in the 
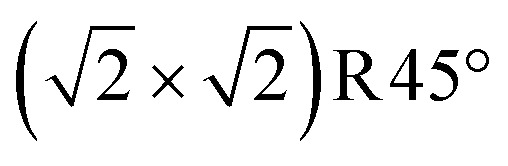
 unit cell, in both the surface and the first subsurface Fe_oct_ layer. Fig. 2 shows that the structure in Fig. 6b is more stable than that in Fig. 6a in r^2^SCAN+U calculations, while the two structures are almost degenerate in PBE+U calculations (Fig. S8†). The structure of Fig. 6a was previously proposed by Novotny *et al.*^26^ Similar to the structure of Fig. 5a it suffers from repulsion between the tetrahedral Fe atoms and the added Fe atoms (Fe_B*_), with a distance of only ≈2.6 Å. Thus, the row of additional Fe_B*_ atoms, shown in cyan (spin down), is not coplanar with the other Fe_oct_ atoms of the surface but 0.5 Å above the Fe_B_ atoms of the bulk truncated surface (see the perspective view in Fig. S5j†). The other model with the same stoichiometry (Fig. 6b) is an FeO-like termination obtained by adding two FeO layers to the DBT surface of Fig. 3b. This results in the outermost three layers of the slab made from Fe with octahedral coordination. Since Fig. 5c has a surplus of one Fe atom with respect to a stoichiometric surface and the added layer has a surplus of 2 Fe atoms (Fe_8_O_8_*vs.* the stoichiometric Fe_6_O_8_) per 
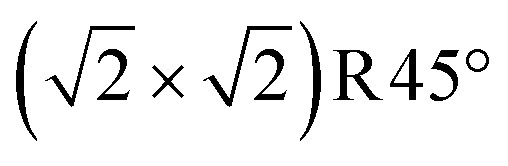
 cell, this structure also corresponds to an Fe excess of 3 atoms. In the relaxed structure, the surface Fe atoms are coplanar. The DFT calculations show only Fe^2+^ in the first layer. In spite of its FeO-like structure, the second layer contains both Fe^2+^ and Fe^3+^. The calculated Bader charges are reported in Table S7.† As all the other structures, this termination has antiparallel spins between neighboring Fe rows. Within a (001) layer, this is the same as expected for FeO(001). Concerning the spin arrangement of adjacent layers, the subsurface layer in Fig. 6b has the spin-down rows running parallel to those in the first layer. This configuration is the same as the bulk spin order of FeO, where the spins are parallel within {111} planes, but adjacent {111} planes have opposite spin. There is also an alternative configuration in which the spin-down rows of the surface and subsurface layers are oriented at a 90° angle. This configuration has surface energy that is almost degenerate with the one where spin-down rows run parallel to those in the first layer. As mentioned above, the subsurface layer of the termination in Fig. 6(b) is not purely Fe^2+^, thus it differs from bulk FeO.

Similar to the 1 layer FeO-like termination, surface rumpling of oxygen is also observed for 2 layers FeO but is less pronounced (see Fig. S5k†). In the current case, all oxygen atoms in the surface layer are 5-fold coordinated to Fe_B_ atoms, with one bond to an Fe_B_ directly beneath. The rumping is the result of the repulsion between the oxygens atoms and the differently oriented t_2g_ orbitals of the Fe^2+^ ions in the subsurface layer. Furthermore, the interaction between the t_2g_ orbitals of the Fe^2+^ atoms in the surface layer and the p-orbitals of the oxygen atoms leads to in-plane displacements, as shown in Fig. S7.†

## Discussion

Our updated phase diagram for the Fe_3_O_4_(001) surface (Fig. 2) predicts two stable surfaces in addition to the SCV termination at the r^2^SCAN+U level. Starting from the well-characterized oxygen-rich SCV termination,^17,18^ the next stable structure as the conditions become more reducing is the stoichiometric Fe_oct_ pair (or Fe-dimer) termination. This surface is already well known for STM experiments with varying explanations, but here we show that the lowest-energy structure is that proposed by Rustad *et al.*^28^ The Fe coverage of this structure (2 Fe atoms per 
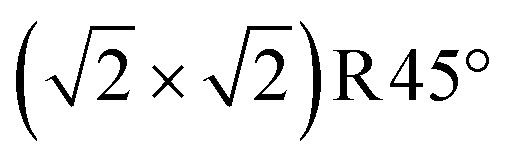
 cell in addition to the SCV structure) is consistent with recent experiments;^19^ also the simulated STM image (Fig. S6†) agrees well with the experimental STM data.^19,20,26,27^ It is interesting to note that this model was not considered in more recent attempts to establish the surface phase diagram of Fe_3_O_4_(001).^26,49,52,60^ Although this structure is stoichiometric, it also makes sense as a first step towards reducing the surface region towards FeO, which contains only octahedrally coordinated Fe^2+^. Essentially, Rustad's Fe_oct_-pair structure replaces one subsurface Fe_A_^3+^ of the bulk-truncated magnetite surface with two octahedral Fe atoms. Interestingly, the new cations in the Fe_oct_ pair are both Fe^3+^, and the nearby Fe_B_ atoms are reduced to Fe^2+^. This can be rationalized because all surface Fe atoms have the same 5-fold oxygen coordination and four Fe neighbors in the first coordination shell, but next-nearest Fe are different: the Fe_B_ atoms have an Fe_A_ neighbor in the first subsurface layer at a distance of ≈3.35 Å, while the atoms of the Fe_oct_ pair have a much larger distance to the Fe_A_ (4.6 Å). The average charge of the surface layer 
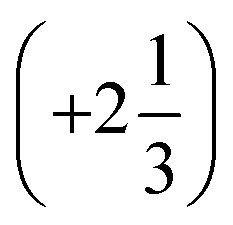
 differs from the nominal 2.5+ of octahedral layers in magnetite. The same is true for the competing 0.5 ML Fe_A*_ model (Table S4†). We consider it likely that it is more favorable to have the Fe^2+^ with its occupied minority-spin t_2g_ orbital in the surface layer, where the repulsive overlap of this orbital and the neighboring oxygen atoms is lower than in a fully oxygen-coordinated bulklike site of the subsurface Fe_B_ layer.

As expected for superexchange favoring antiferromagnetic alignment of Fe atoms coupled *via* common oxygen neighbors, the surface Fe_B_ rows from the Fe_3_O_4_ and the new surface Fe_oct_ cations have antiparallel spin (the same is true for all the similar structures, also with more Fe-rich stoichiometry). The substitution of one Fe_A_ with two Fe_B_ comes at the expense of some surface strain, however: converting Fe_3_O_4_ into FeO would require a 2.1% lattice expansion according to the r^2^SCAN+U-calculated lattice constants. Thus, the surface Fe_B_ cations relax by 0.08 Å away from the Fe_oct_ pair to accommodate it in the surface. Moreover, two O atoms neighboring the Fe_oct_ pair move up, which also helps to relieve stress. This upward buckling may be also related to the fact that oxygen usually does not prefer a planar 4-fold geometry.

The relative stability of Rustad's Fe_oct_ pair termination can also be rationalized in terms of electronic and structural effects. In a purely ionic model, Fe_3_O_4_(001) is a polar surface with alternating planes of ±6 *e* per 
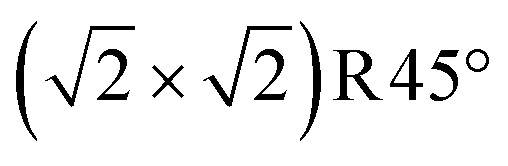
 cell in the 〈001〉 directions (Fig. S10†). In principle, compensation of the macroscopic dipole of the B-termination (DBT) or a 1 ML Fe_A_ termination requires that the surface region has a net charge of +3 or −3 *e*, respectively. Indeed, this is why the 0.5 ML Fe_A_ model was originally proposed; terminating the DBT surface with a single Fe_A_ atom per unit cell would provide the necessary +3 charge.^63,64^ However, utilizing integer charges as in a truncated bulk is overly simplistic in a half-metallic, multivalent oxide with delocalized charges, and our calculations suggest that a hypothetical twofold coordinated surface Fe_A_ would be actually Fe^2+^-like (not Fe^3+^ as Fe_A_ in the bulk) and also the Fe_B_ surface layer would not retain its average 2.5+ state (Fig. S2a†); the additional charge is localized in the subsurface Fe_B_ atoms. The Fe_oct_ pair proposed by Rustad has the same overall stoichiometry, but is more favorable because it avoids highly undercoordinated metal atoms.^45^ All Fe in the surface layer is fivefold coordinated, and the Fe_oct_-pair atoms bind down to the subsurface O atoms that have lost an Fe_A_ bonding partner, ensuring these remain fourfold coordinated.

As we move to reducing conditions, the next surface to consider has one excess Fe atom per 
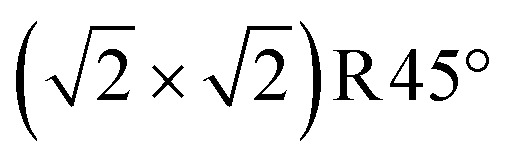
 cell with respect to the stoichiometric surface. Different functionals predict different lowest-energy terminations, but neither of them is on the convex hull and therefore expected to occur near equilibrium. One structure with this stoichiometry is the 1-layer FeO termination (Fig. 5c). This surface features two Fe_oct_ pairs instead of the Fe_A_ atoms present in a truncated bulk. It can be also considered a single layer of FeO added to a truncated bulk, and, therefore, a first step toward the FeO-like termination. Another structure with this stoichiometry has a lower energy in PBE calculations; it has two Fe_A*_ adatoms near octahedral sites on a truncated-bulk substrate (Fig. 5b). In contrast to the FeO monolayer, this structure can avoid the problem of ≈2% lattice mismatch between an FeO layer and the underlying Fe_3_O_4_ substrate by slight lateral undulation of the Fe_B_ rows; it also avoids fourfold planar coordination of oxygen. This comes at the cost of repulsive interactions of the Fe_A*_ surface atoms with a subsurface Fe_A_ (2.5 Å distance).

In extremely reducing conditions, the last stable surface predicted in this study is terminated by three fully octahedral FeO(001)-like layers (Fig. 6b). This structure can be considered the result of adding one FeO layer to the 1-layer FeO of Fig. 5c. In this structure, all of the Fe in the topmost surface layer is Fe^2+^, and the antiferromagnetic alignment is as expected for FeO(001). In principle, this process can occur deeper and deeper into the bulk, and it likely underlines the overall transformation from Fe_3_O_4_ to FeO. A defective and rumpled version of the FeO termination has been observed previously by scanning tunneling microscopy.^26^

For most structures, the spin orientations of the Fe atoms can be predicted because superexchange *via* the O anions leads to antiferromagnetic coupling of neighboring Fe rows. Therefore, it is clear that the Fe atoms within Rustad's Fe_oct_ pair surface must be antiparallel to the Fe_B_ in the bulk truncated surface and the Fe_B_ in the layer below. For comparison, we find that the Fe_oct_ pair termination with the same Fe spin direction as the neighboring Fe_B_ in the surface layer is less stable by 4.40 meV Å^−2^ (Table S8†). We also note that the charge order of Fe^2+^-like and Fe^3+^-like ions in the surface layer has a substantial influence on the energy (Fig. S3 and S4†); the influence of the charge states of the Fe_oct_ in the subsurface layers is less pronounced. As a general point, our work demonstrates that structural relaxation in DFT+U calculations does not guarantee obtaining the electronic ground state solution in a mixed-valency system.

The conditions required to form the reduced surface structures appear to be unrealistically reducing at the typical temperatures used in experiments (see the top *x* axes of Fig. 2). However, experimentally, the FeO termination was observed under non-equilibrium conditions, either through sputtering with Ar^+^ ions (a procedure that preferentially removes surface O atoms) or by deposition of metallic Fe. In the case of experiments involving thin films, reduced terminations were reported when the Fe_3_O_4_(001) film was deposited on an Fe buffer layer.^20,22,24,27,58,62,65^ This excess Fe would diffuse into the film at the growth conditions or upon annealing; the presence of metallic Fe is again incompatible with the assumption of thermodynamic equilibrium of the oxide with the O_2_ gas phase and indicates that the conditions are more reducing than what one would expect from the O_2_ pressure in the experiment.

While the SCV and 2-layer FeO structures are at the convex hull of the surface phase diagram, their stability ranges do not overlap with the bulk stability range of Fe_3_O_4_ in the calculated phase diagram (Fig. 2). Taking the phase diagram at face value, this means that the conditions required to from the SCV would be also sufficiently oxidizing to convert the bulk into Fe_2_O_3_, and the conditions required for forming the 2-layer FeO termination would also convert the bulk into FeO when kinetic limitations are absent. In practice, this is not a limitation for experiments, since the typical temperatures and time scales in surface science experiments are usually far from those required for a bulk phase transition. It must be also noted that the exact conditions for phase transitions are not only determined by the DFT energies at *T* = 0, as Fig. 2 would suggest. At finite temperatures, vibrational energy and entropy as well as other terms (*e.g.* entropy from different charge configurations with similar energy, vacancies, defects) have to be considered and may change the relative stabilities of the structures, especially if the energy differences are small and the competing phases differ strongly in their properties.

In spite of these limitations, our revised surface phase diagram of Fe_3_O_4_(001) provides valuable insights into the structural evolution and stability of iron oxide surfaces under reducing conditions. The agreement between computational results and experimental observations in the literature underlines the reliability of the proposed surface models, paving the way for further exploration of Fe_3_O_4_(001) surfaces under reducing conditions in diverse applications.

## Conclusion

Our revised surface energy phase diagram of the Fe_3_O_4_(001) shows that the model originally proposed by Rustad *et al.* is the most stable stoichiometric structure. Rustad's model represents a first step toward the wüstite structure of FeO, because it replaces tetrahedrally coordinated Fe_A_ atoms with octahedrally coordinated Fe atoms. Additionally, we found that the charge states of the Fe_B_ atoms in the surface layers play a crucial role in determining surface stability. More broadly, our calculations indicate that the mere structural relaxation in DFT+U calculations does not necessarily ensure convergence to the electronic ground state for Fe_3_O_4_(001) surfaces.

## Data availability

All computational structures and relevant data generated in this study are available in the ESI.†

## Conflicts of interest

There are no conflicts to declare.

## Supplementary Material

LF-002-D5LF00022J-s001

LF-002-D5LF00022J-s002

LF-002-D5LF00022J-s003

LF-002-D5LF00022J-s004

LF-002-D5LF00022J-s005

LF-002-D5LF00022J-s006

LF-002-D5LF00022J-s007

LF-002-D5LF00022J-s008

LF-002-D5LF00022J-s009

LF-002-D5LF00022J-s010

LF-002-D5LF00022J-s011

LF-002-D5LF00022J-s012
