# Supplementary material for: The surface phase diagram of Fe3O4(001) revisited
Source: RSC Appl Interfaces. 2025 Mar 14;2(3):673–83. doi: 10.1039/d5lf00022j (PMC11947718; doi:10.1039/d5lf00022j)
Supplement: LF-002-D5LF00022J-s004 [file LF-002-D5LF00022J-s004.pdf]

Supplementary Information (SI) for RSC Applied Interfaces.
This journal is © The Royal Society of Chemistry 2025

Supplementary Information

The Surface Energy Diagram of Fe_3O_4(001) Revisited

Panukorn Sombut[1], Matthias Meier[1,2], Moritz Eder[1], Thomas Angerler[1], Oscar Gamba[3], Michael Schmid[1], Ulrike Diebold[1], Cesare Franchini[2,4] and Gareth S. Parkinson[1]*

[1]Institute of Applied Physics, TU Wien, Vienna, Austria

[2]Faculty of Physics, Center for Computational Materials Science, University of Vienna, Vienna, Austria

[3]GeoRessources, Université de Lorraine, CNRS, 54000, Nancy, France

[4]Dipartimento di Fisica e Astronomia, Università di Bologna, Bologna, Italy

The PDF file includes:

Figures S1 to S9

Table S1 to S8

Other Supplementary Material for this manuscript includes the following:

"cif" files for the optimized structure models present in the paper at r^2SCAN+U level:

1.) SCV
2.) DBT
3.) Fe_A on SCV
4.) V_O in SCV
5.) Fe_{oct} pair (Rustad)
6.) 0.5 ML Fe_{A*} on DBT
7.) 0.5 ML Fe_{oct} pair on DBT
8.) 1 ML Fe_{A*} on DBT
9.) 1 layer FeO-like
10.) 1 ML Fe_{oct} pair on DBT
11.) 2 layers FeO-like

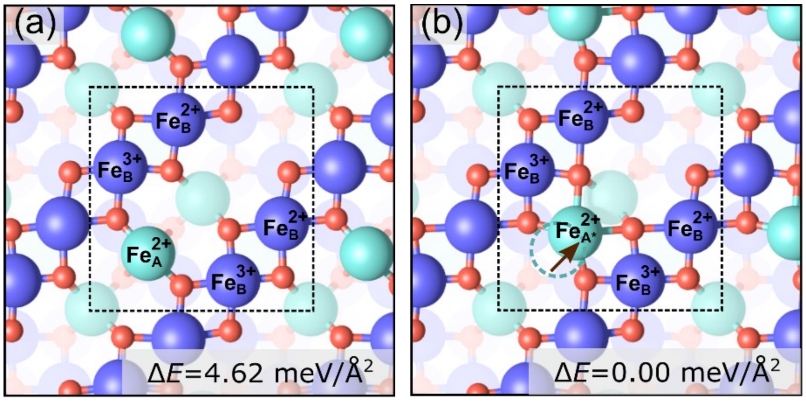

Figure S1. Relaxation of the 0.5 ML Fe_{A*} termination (top view): In the DFT calculations, the Fe_A atom relaxes away from its high-symmetry position, with an energy gain of 4.62 meV/Å^2, which corresponds to 0.33 eV per (√2×√2)R45° surface cell. (The conversion factor is given by the size of the surface cell, 70.76 Å^2). (a) The initial configuration with relaxation in z only, and (b) the result of the full relaxation. ΔE is the relative surface energy difference with respect to the ground-state configuration. Fe is blue and cyan (large), and O is red (small). Dark blue (spin up) and cyan (spin down) indicate the spin orientation in Fe atoms. A (√2×√2)R45 unit cell is indicated with a black dashed square.

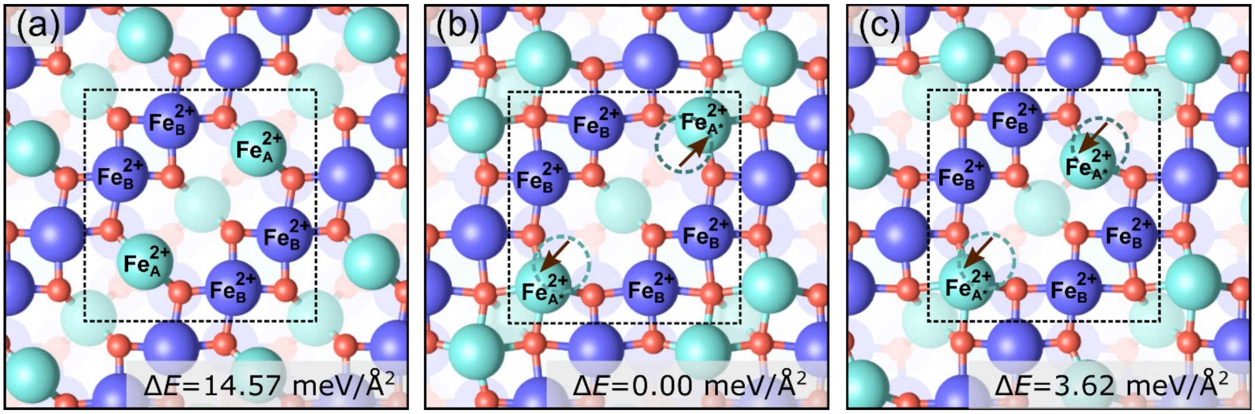

Figure S2. **1 ML Fe_{A*} termination (top view):** In the DFT calculations, the Fe_A atoms relax away from their high-symmetry position in the opposite direction, associated with an energy gain of 14.57 meV/Å^2, which corresponds to a total energy of 1.03 eV per (√2×√2)R45° surface cell. (a) The initial configuration with relaxation in z only, (b) the configuration obtained after full DFT relaxation, where the Fe_{A*} atoms relax in opposite directions, and (c) the configuration obtained after full DFT relaxation, where the Fe_{A*} atoms relax in the same direction. ΔE is the relative surface energy difference with respect to the ground-state configuration.

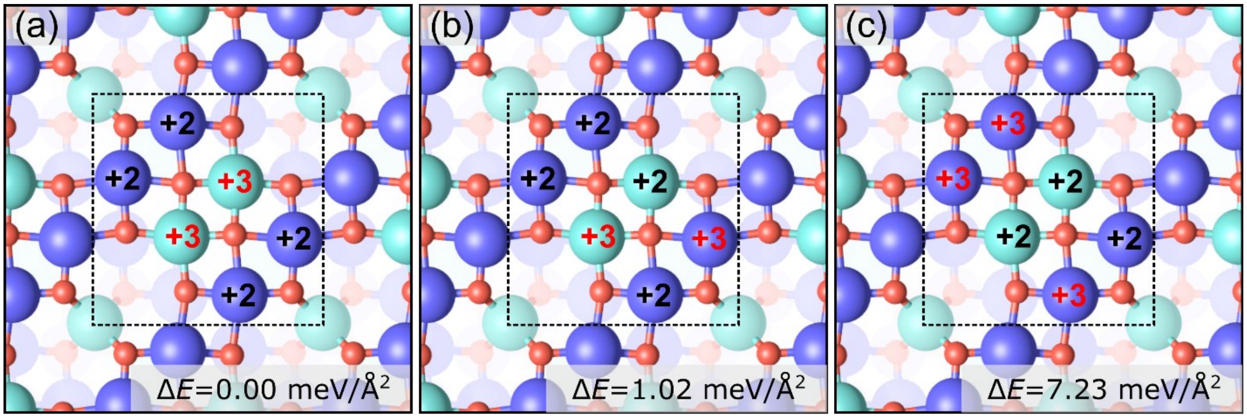

Figure S3. **Effect of charge ordering on the surface stability of Rustad's Fe_{oct} pair termination:** Different electronic configurations of the Fe_{oct} pair termination obtained at the r^2SCAN+U level. The assignment of the Fe charges, 2+ and 3+, is based on the calculated Bader charges of 1.30−1.45 *e* and 1.70−1.80 *e*, respectively. Here we show three electronic configurations of the Fe_{oct} pair termination. Comparison of the energies for three different electronic configurations shows that the charge ordering in the topmost layer plays a significant role in the surface stability: The energy difference of 7.23 meV/Å^2 corresponds to a total energy of 0.51 eV per $(\sqrt{2}\times\sqrt{2})R45°$ surface cell. Different of charge ordering in the subsurface layer causes comparably small variations of the surface energy (less than 1 meV/Å^2). Without setting the initial orbital occupations, both DFT functionals, r^2SCAN+U and PBE+U, as well as a hybrid functional (HSE06) got stuck in a local minimum, configuration (c). To counteract this, we used the occupation matrix tool to control the charge order of the Fe atoms in the surface layer. ΔE is the relative surface energy difference with respect to the ground-state configuration.

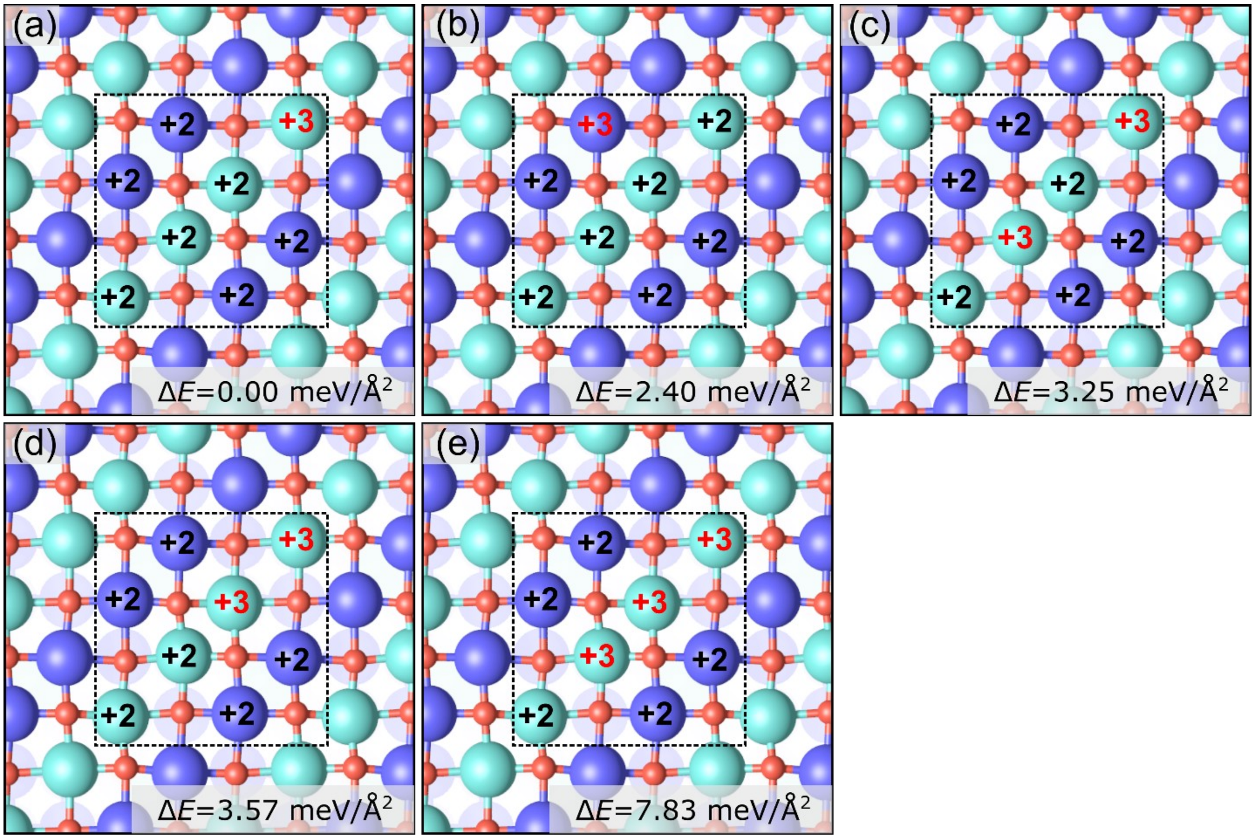

Figure S4. **Effect of charge ordering on the surface stability on the 1 layer FeO-like termination:** Different electronic configurations of the FeO-only termination obtained at the r^2SCAN+U level. Fe is blue and cyan (large), and O is red (small). Dark blue (spin up) and cyan (spin down) indicate the spin orientation in Fe atoms. A (√2×√2)R45 unit cell is indicated with a black dashed square. The assignment of the Fe charges, 2+ and 3+, is based on the calculated Bader charges of 1.30−1.45 *e* and 1.70−1.80 *e*,, respectively. Here we show five electronic configurations of the FeO-only termination. Comparison of the energies for five different electronic configurations shows that the charge ordering in the topmost layer plays a significant role in the surface stability: The energy difference of 7.83 meV/Å^2 corresponds to a total energy of 0.55 eV per (√2×√2)R45° surface cell. The difference of charge ordering in the subsurface layer causes comparably small variations of the surface energy (less than 1 meV/Å^2). ΔE is the relative surface energy difference with respect to the ground-state configuration.

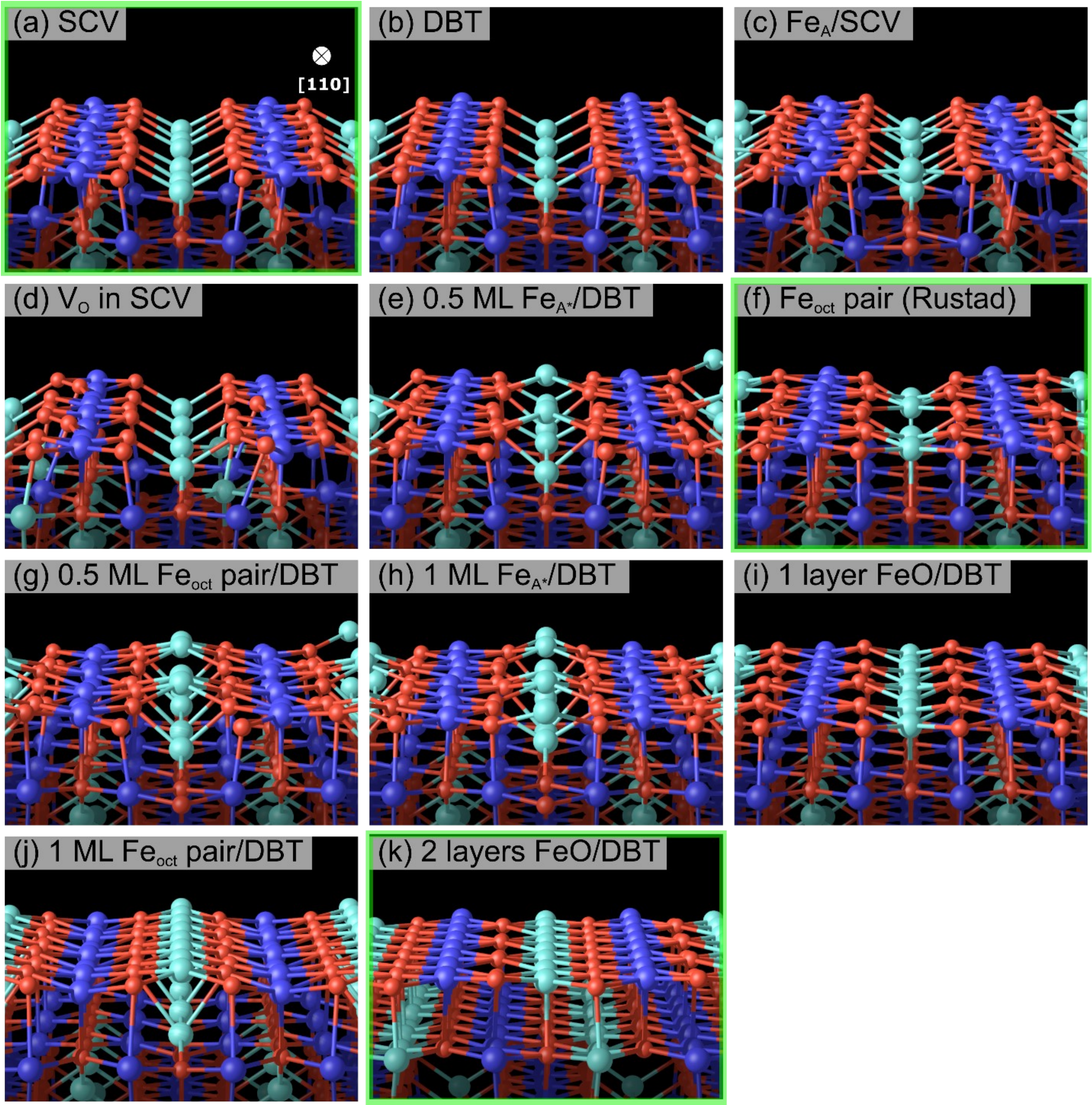

Figure S5. **Various terminations of the Fe_3O_4(001) facet (perspective view).** (a) SCV, (b) DBT, (c) Fe_A on SCV, (d) V_O in SCV, (e) 0.5 ML Fe_{A*} on DBT, (f) Fe_{oct} pair proposed by Rustad, (g) 0.5 ML Fe_{oct} pair on DBT proposed by Novotny *et al*., (h) 1 ML Fe_{A*} on DBT, (i) 1 layer FeO, (j) 1 ML Fe_{oct} pair on DBT proposed by Novotny *et al*., and (k) 2 layers FeO (spin rows ||). Fe is blue and cyan (large), and O is red (small). Dark blue (spin up) and cyan (spin down) indicate the spin orientation in Fe atoms. The highlighted green squares indicate the three stable terminations in the convex hull of the surface phase diagram for Fe_3O_4(001) at the r^2SCAN+U level.

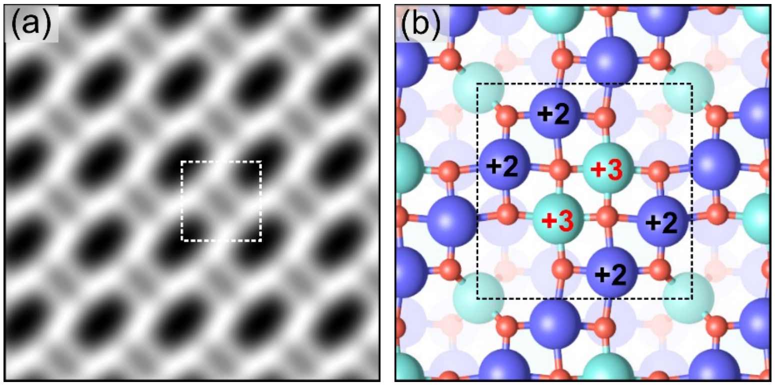

Figure S6. **The Fe_{oct} pair termination proposed by Rustad *et al.***[3] (a) Simulated STM images were created by using the Tersoff-Hamann approximation[4] in constant-height mode at a height of 4 Å above the first-layer atom centers, and (b) optimized structure of Rustad's model (top view). Fe is blue and cyan (large), and O is red (small). Dark blue (spin up) and cyan (spin down) indicate the spin orientation in Fe atoms. A (√2×√2)R45 unit cell is indicated with white and black dashed squares.

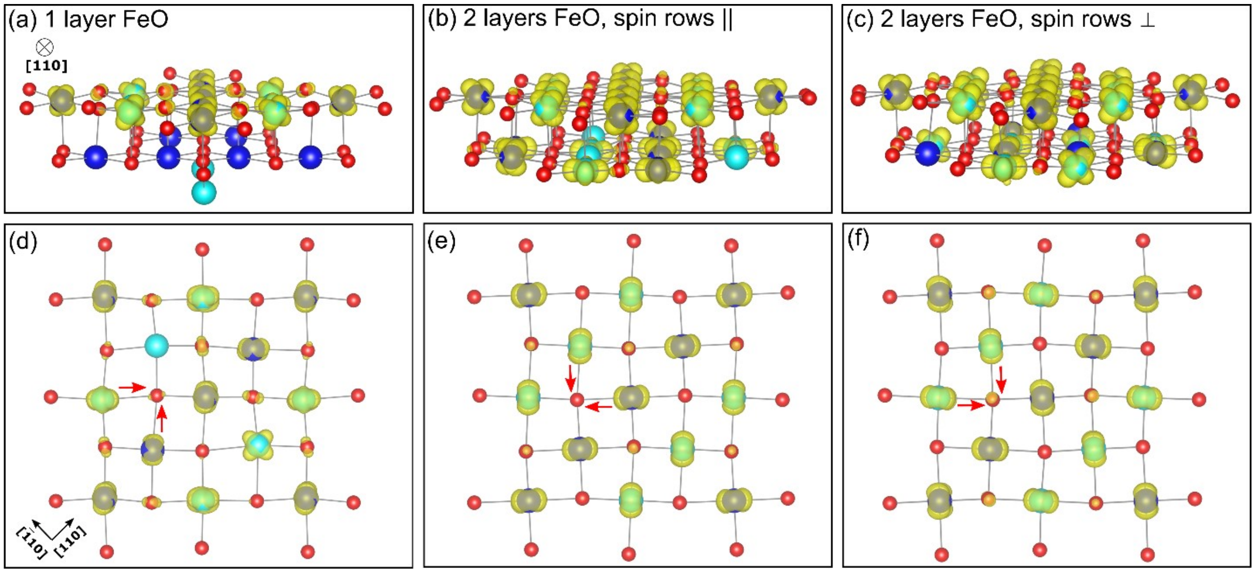

Figure S7. **Orbitals of 1 layer and 2 layers FeO-like on DBT**. (a–c) perspective view and (d–f) top view of (a,d) 1 layer FeO-like, (b,e) 2 layers FeO-like, with spin-down rows in the subsurface layer running parallel to those in the surface layer, and (c,f) 2 layers FeO-like, with the spin-down rows of the surface and subsurface layers perpendicular to each other. All Fe atoms in the surface layer of 2 layers FeO-like are Fe^{2+} ions. The t_{2g} orbitals of the partially occupied spin channel of Fe^{2+} are shown in the figures. Iron (Fe) is large, blue (spin up) or cyan (spin down), and oxygen (O) is small and red. The red arrows indicate the repulsion between the Fe t_{2g} orbitals and the oxygen, which causes the in-plane displacement of the oxygen atoms. The out-plane displacements of the oxygen atoms in 2 layers FeO-like on DBT arise from the interaction with Fe t_{2g} (d_{xz} and d_{yz}) in the subsurface layer, whereas in the 1 layer FeO-like, the displacements are due to a 4-fold planar coordination. These figures were created using VESTA[5].

Table S1: SCV termination

The local spin magnetic moments M (in μ_B) for the outermost five layers of the SCV termination are determined in DFT calculations (r^{2}SCAN+U). We assigned Fe^{2+}-like and Fe^{3+}-like cations by the calculated Bader charge of 1.35-1.50 e and 1.70-1.85 e, respectively. Fe^{2+} can be also recognized by the lower value of $|M|$ (below 4 μ_B). The surface layer is denoted by "S", the layer below by S − 1, etc. The sequence of the atoms within the layers is the same as in the .cif files.

Layer		Bader charge (e)	M (μ_B)
S	Fe_B	1.78	4.227
S	Fe_B	1.78	4.227
S	Fe_B	1.79	4.226
S	Fe_B	1.79	4.226
S − 1	Fe_A	1.77	−4.180
S − 1	Fe_A	1.77	−4.179
S − 1	Fe_{int}	1.75	−4.177
S − 2	Fe_B	1.84	4.243
S − 2	Fe_B	1.84	4.240
S − 3	Fe_A	1.74	−4.114
S − 3	Fe_A	1.74	−4.114
S − 4	Fe_B	1.82	4.234
S − 4	Fe_B	1.78	4.252
S − 4	Fe_B	1.78	4.232
S − 4	Fe_B	1.84	4.233

Table S2: DBT

Local spin magnetic moments and Bader charges for the outermost five layers of the DBT in the lowest-energy configuration. See table S1 for more information.

Layer		Bader charge (e)	M (μ_B)
S	Fe_B	1.74	4.234
S	Fe_B	1.74	4.237
S	Fe_B	1.74	4.234
S	Fe_B	1.74	4.237
S − 1	Fe_A	1.78	−4.176
S − 1	Fe_A	1.78	−4.176
S − 2	Fe_B	1.77	4.254
S − 2	Fe_B	1.77	4.254
S − 2	Fe_B	1.77	4.254
S − 2	Fe_B	1.77	4.254
S − 3	Fe_A	1.74	−4.136
S − 3	Fe_A	1.74	−4.136
S − 4	Fe_B	1.77	4.194
S − 4	Fe_B	1.42	3.708
S − 4	Fe_B	1.77	4.194
S − 4	Fe_B	1.42	3.708

Table S3: Fe_{oct} pair termination (Rustad's model)

Local spin magnetic moments and Bader charges for the outermost seven layers of the Rustad's Fe_{oct} pair termination in the lowest-energy configuration. See table S1 for more information.

Layer		Bader charge (e)	M (μ_B)
S	Fe_B	1.40	3.706
S	Fe_B	1.40	3.709
S	Fe_B	1.39	3.706
S	Fe_B	1.40	3.709
S	Fe_{oct} pair	1.77	−4.217
S	Fe_{oct} pair	1.77	−4.217
S − 1	Fe_A	1.76	−4.173
S − 2	Fe_B	1.79	4.246
S − 2	Fe_B	1.79	4.246
S − 2	Fe_B	1.77	4.242
S − 2	Fe_B	1.77	4.242
S − 3	Fe_A	1.76	−4.150
S − 3	Fe_A	1.76	−4.150
S − 4	Fe_B	1.76	4.234
S − 4	Fe_B	1.78	4.187
S − 4	Fe_B	1.39	3.733
S − 4	Fe_B	1.78	4.237
S – 5	Fe_A	1.74	−4.155
S – 5	Fe_A	1.74	−4.155
S – 6	Fe_B	1.42	3.709
S – 6	Fe_B	1.42	3.709
S – 6	Fe_B	1.42	3.718
S – 6	Fe_B	1.41	3.719

Table S4: 0.5 ML Fe_{A*} on DBT

Local spin magnetic moments and Bader charges for the outermost seven layers of the 0.5 ML Fe_{A*} on DBT in the lowest-energy configuration. See table S1 for more information.

Layer		Bader charge (e)	M (μ_B)
S + 1	Fe_{A*}	1.34	−3.696
S	Fe_B	1.39	3.724
S	Fe_B	1.73	4.210
S	Fe_B	1.40	3.718
S	Fe_B	1.73	4.212
S − 1	Fe_A	1.71	−4.127
S − 1	Fe_A	1.78	−4.175
S − 2	Fe_B	1.80	4.262
S − 2	Fe_B	1.74	4.211
S − 2	Fe_B	1.80	4.238
S − 2	Fe_B	1.80	4.261
S − 3	Fe_A	1.74	−4.135
S − 3	Fe_A	1.74	−4.138
S − 4	Fe_B	1.41	3.717
S − 4	Fe_B	1.78	4.205
S − 4	Fe_B	1.45	3.743
S − 4	Fe_B	1.77	4.204
S – 5	Fe_A	1.74	−4.156
S – 5	Fe_A	1.75	−4.156
S – 6	Fe_B	1.43	3.717
S – 6	Fe_B	1.41	3.714
S – 6	Fe_B	1.41	3.714
S − 6	Fe_B	1.41	3.717

Table S5: 1 ML Fe_{A*} on DBT

Local spin magnetic moments and Bader charges for the outermost five layers of the 1 ML Fe_{A*} on DBT in the lowest-energy configuration. See table S1 for more information.

Layer		Bader charge (*e*)	M (μ_B)
S + 1	Fe_{A*}	1.34	−3.714
S + 1	Fe_{A*}	1.34	−3.716
S	Fe_B	1.39	3.698
S	Fe_B	1.38	3.700
S	Fe_B	1.37	3.698
S	Fe_B	1.40	3.706
S − 1	Fe_A	1.76	−4.173
S − 1	Fe_A	1.63	−4.079
S − 2	Fe_B	1.77	4.224
S − 2	Fe_B	1.80	4.246
S − 2	Fe_B	1.78	4.246
S − 2	Fe_B	1.78	4.243
S − 3	Fe_A	1.73	−4.139
S − 3	Fe_A	1.73	−4.143
S − 4	Fe_B	1.46	3.724
S − 4	Fe_B	1.76	4.194
S − 4	Fe_B	1.42	3.719
S − 4	Fe_B	1.77	4.201

Table S6: 1 layer FeO on DBT

Local spin magnetic moments and Bader charges for the outermost four layers of the FeO-only on DBT in the lowest-energy configuration. Note that there is no tetrahedral S – 1 layer in this structure; thus the S – 1 layer (Fe_B) corresponds to the S – 2 layers of the previous tables, and also the indices of the deeper layers are shifted by one. See table S1 for more information.

Layer		Bader charge (*e*)	M (μ_B)
S	Fe_B	1.38	3.713
S	Fe_B	1.38	3.715
S	Fe_B	1.38	3.709
S	Fe_B	1.37	3.702
S	Fe_{B*}	1.39	−3.706
S	Fe_{B*}	1.37	−3.682
S	Fe_{B*}	1.71	−4.199
S	Fe_{B*}	1.41	−3.707
S − 1	Fe_B	1.77	4.261
S − 1	Fe_B	1.75	4.261
S − 1	Fe_B	1.78	4.267
S − 1	Fe_B	1.76	4.255
S − 2	Fe_A	1.76	−4.159
S − 2	Fe_A	1.76	−4.159
S − 3	Fe_B	1.75	4.248
S − 3	Fe_B	1.77	4.219
S − 3	Fe_B	1.41	3.741
S − 3	Fe_B	1.74	4.194

Table S7: 2 layers FeO on DBT

Local spin magnetic moments and Bader charges for the outermost three layers of the 2 layers of FeO on DBT (spin rows ⊥) in the lowest-energy configuration. See table S1 for more information.

Layer		Bader charge (*e*)	*M* (μ_B)
S	Fe_B	1.35	3.704
S	Fe_B	1.34	3.699
S	Fe_B	1.35	3.704
S	Fe_B	1.34	3.703
S	Fe_{B*}	1.35	−3.695
S	Fe_{B*}	1.35	−3.696
S	Fe_{B*}	1.36	−3.700
S	Fe_{B*}	1.35	−3.694
S − 1	Fe_B	1.39	3.731
S − 1	Fe_B	1.74	4.231
S − 1	Fe_B	1.39	3.760
S − 1	Fe_B	1.75	4.217
S − 1	Fe_{B*}	1.37	−3.676
S − 1	Fe_{B*}	1.39	−3.691
S − 1	Fe_{B*}	1.37	−3.677
S − 1	Fe_{B*}	1.39	−3.687
S − 2	Fe_B	1.74	4.222
S − 2	Fe_B	1.78	4.249
S − 2	Fe_B	1.76	4.233
S − 2	Fe_B	1.41	3.724

Table S8: Effect of spin orientation on the surface energy for the Fe_{oct} pair and 2-layer FeO terminations.

The arrows indicate the spin orientation of the Fe atoms in the surface layer. In the bulk, Fe_A atoms have spin down (↓) and Fe_B have spin up (↑). ΔE is the relative energy with respect to the most stable configuration. We note that the spin orientation of the 2 layers FeO structure in the S − 1 layer has the spin rows parallel to the spin row in the surface layer. The "↓↑" stands for the Fe_{B*} pair atoms have alternating spins in the row.

	**Fe_B**	**Fe_{oct} pair**	**ΔE (meV/Å^2)**
Fe_{oct} pair (Rustad)	↑	↓↓	+0.00
	↑	↑↑	+4.40
2 layers of FeO on DBT	Fe_B	Fe_{B*}	
	↑	↓↓	+0.00
	↑	↓↑	+5.48
	↑	↑↑	+7.96

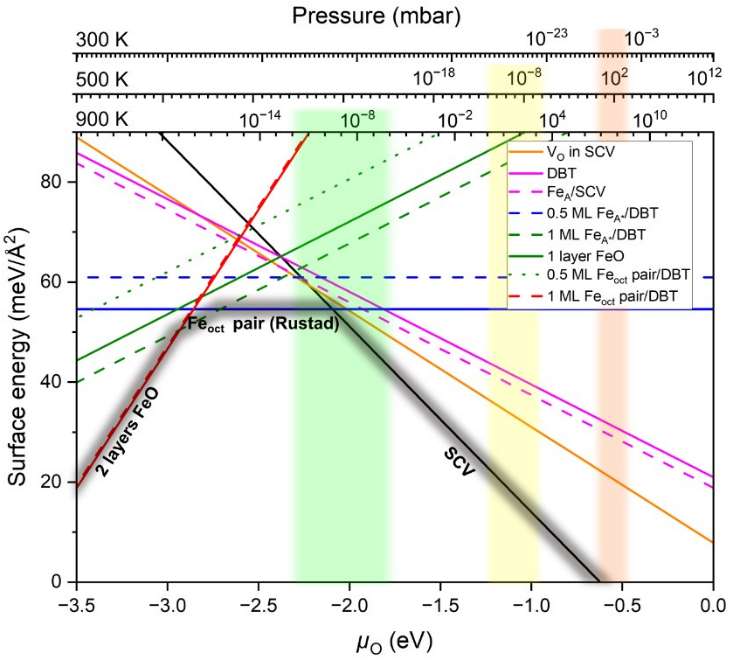

Figure S8. **Surface phase diagram of Fe_3O_4(001) computed with PBE+U with the DFT lattice constant.** See Fig. 2 of the main text for further explanations.

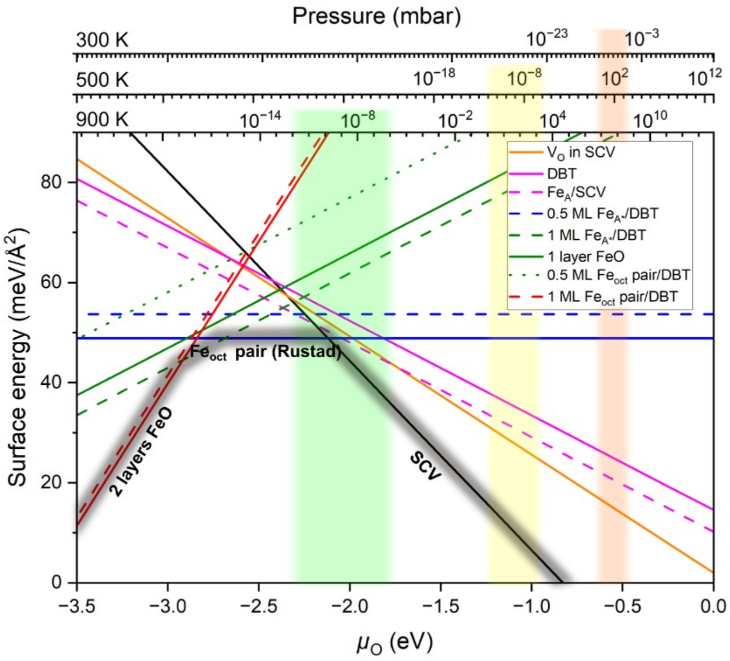

Figure S9. **Surface phase diagram of Fe_3O_4(001) computed with PBE+U with the experimental lattice constant.** See Fig. 2 of the main text for further explanations. These calculations were done with an asymmetric slab (10 octahedral and 9 tetrahedral layers, bottom 11 layers fixed, 14 Å vacuum).

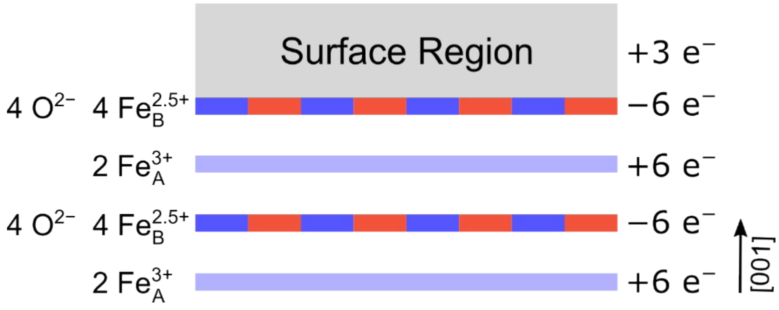

Figure S10. Schematic illustration of polarity compensation at the Fe_3O_4(001) surface. Along the (001) direction, Fe_3O_4 consists of alternating layers containing two Fe_A cations and a plane comprising eight O^{2-} anions and four Fe_B cations, with an average charge state of +2.5. As a result, the A and B layers have a net charge of +6 and −6 electrons, respectively. This would make the bulk-truncated, B-terminated Fe_3O_4(001) a polar surface. To satisfy electrostatic stability in a simple ionic model, compensation requires adding a + 3 charge in the surface region, e.g. by adding one Fe^{3+} atom per (√2×√2)R45° cell.

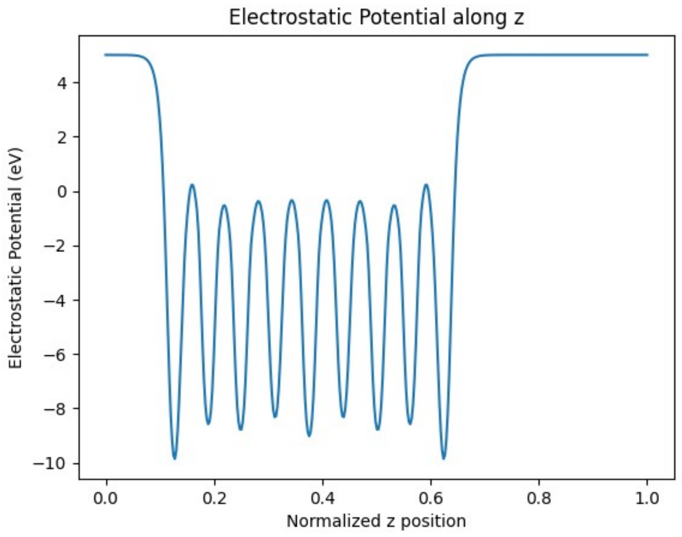

Figure S11. Projection of electrostatic potential along the normalized z-direction for the Fe_{oct} pair (Rustad) termination. The oscillations in the potential reflect the atomic-layer-dependent variations within the slab, while the converged plateau regions at both surfaces indicate the vacuum level. The absence of a slope (electric field) in the vacuum and the symmetry with respect to the slab center shows that the (slight) symmetry breaking by the low-temperature phase does not cause a dipole moment that may affect the accuracy of the calculations.

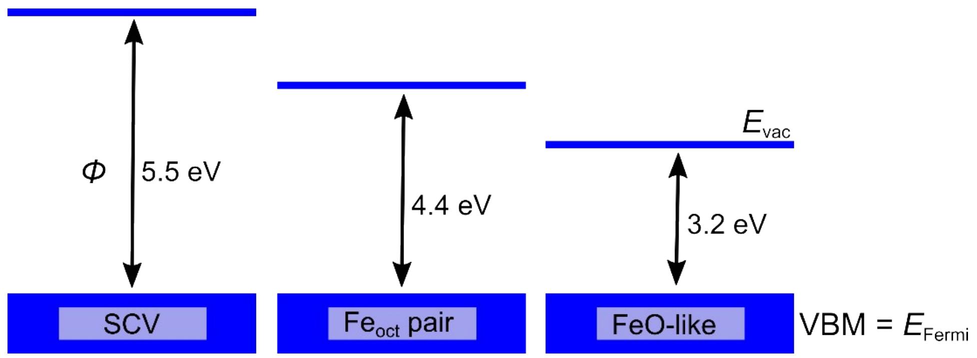

Figure S12. A schematic illustration of the work function (Φ) for the three stable surface terminations on the convex hull, as shown in Figure 2. Note that the calculations do not account for additional effects such as band bending or doping. The work function is computed using the relation: $\Phi = E_{vac} - E_{Fermi}$. The calculated band gaps are ≈ 0.6 eV, 0.6 eV, and 0.9 eV for the SCV termination, the Fe_{oct} pair (Rustad) termination, and the FeO-like termination, respectively. Since the position of the Fermi energy in the band gap is not known, we assume that it coincides with the valence band maximum (VBM), as obtained from the VASP output file. The experimental work function for the Fe_3O_4(001) SCV surface, reported in Reference 6, is 5.20 ± 0.15 eV.

References

(1) Reuter, K.; Scheffler, M. Composition, Structure, and Stability of RuO_2(110) as a Function of Oxygen Pressure. *Phys. Rev. B* **2002**, *65* (3), 1–11. https://doi.org/10.1103/PhysRevB.65.035406.

(2) Rossini, F. D. JANAF Thermochemical Tables. *The Journal of Chemical Thermodynamics*. 1972, pp 509–510. https://doi.org/10.1016/0021-9614(72)90036-5.

(3) Rustad, J. R.; Wasserman, E.; Felmy, A. R. A Molecular Dynamics Investigation of Surface Reconstruction on Magnetite (001). *Surf. Sci.* **1999**, *432* (1–2). https://doi.org/10.1016/S0039-6028(99)00581-6.

(4) Tersoff, J.; Hamann, D. R. Theory of the Scanning Tunneling Microscope. *Phys. Rev. B* **1985**, *31* (2), 805–813. https://doi.org/10.1103/PhysRevB.31.805.

(5) Momma, K. and Izumi, F. VESTA 3 for three-dimensional visualization of crystal, volumetric and morphology data, *J. Appl. Crystallogr.*, **2011**, 44, 1272-1276.

(6) Fonin, M.; Pentcheva, R.; Dedkov, Y. S.; Sperlich, M.; Vyalikh, D. V.; Scheffler, M.; Rüdiger, U.; Güntherodt, G. Surface Electronic Structure of the Fe_3O_4(100): Evidence of a Half-Metal to Metal Transition. *Phys. Rev. B* **2005**, *72* (10), 1–8. https://doi.org/10.1103/PhysRevB.72.104436.
